# Conceptual Framework and Designing for a Seafarers' Health Observatory (SHO) Based on the Centro Internazionale Radio Medico (C.I.R.M.) Data Repository

**DOI:** 10.1155/2020/8816517

**Published:** 2020-12-16

**Authors:** Gopi Battineni, Getu Gamo Sagaro, Nalini Chintalapudi, Francesco Amenta

**Affiliations:** ^1^Telemedicine and Telepharmacy Center, School of Medicinal and Health Sciences Products, University of Camerino, Via Madonna Delle Carceri 9, Camerino 62032, Italy; ^2^Research Department, Centro Internazionale Radio Medico (C.I.R.M.), Via Dell'Architettura 41, Roma 00144, Italy

## Abstract

**Background:**

Health observatory (HO) models are helpful in gathering, analyzing, interpreting, and circulating reliable and quality information on population health and health service delivery. In this study, we proposed an HO conceptual model to enhance seafarer's health, which subjects to disease trends.

**Methods:**

Three methods were followed during the study: a systematic collection of seafarer's health data from the Centro Internazionale Radio Medico (C.I.R.M.) repository, an integrative review of existing seafarer's policy, and both open and closed questionnaires were distributed to stakeholders to develop clinical knowledge. C.I.R.M. is the Italian Telemedical Maritime Assistance Service (TMAS). *Results and Discussion.* A three-layer HO framework was developed, and each layer had its components and functionalities. The proposed HO model integrated with the outcomes of the mentioned methods was working as the origin of the framework. In this way, we can design a standard infrastructure in ships and risk assessment conduction.

## 1. Introduction

The word “observatory” is defined as a framework of monitoring health events and trends with supporting methods [1, 2]. The objective behind the health observatory (HO) is to the supervision of health trends, progressive assessment of medical targets, and provision of evidence in policy and decision-making. Public health observatories are beneficiaries in the supplement of reliable medical data, especially for policymakers that can help to fill gaps related to public health constraints. Moreover, HO's are useful in the assessment of people's health status and system reformation to assist policymakers in the development of holistic and robust health systems of a country [3].

Several countries have developed their own HO's for the general population. The first public HO was established in 1974 by France to provide health information for regional health policy. At present, there are well-organized observatories in France, Belgium, Italy, and later (1990) in England (Liverpool) [4]. Currently, more than sixty observatories were present throughout the world [5], but no health observatory for seafarers was established. Different studies were conducted on seafarers, reported the main causes of mortality and morbidity, and also revealed the source information of seafarers' medical data [6–8]. A study conducted in the USA among seafarers reported that trauma/orthopedic cases were the most frequent type, and cardiovascular diseases (CVD) were the eleventh cause of access to telemedicine service providers. This study concluded that it is impossible to estimate the actual CVD incidence on board without knowing the original population of marine workers [9], which represents knowledge gaps in seafarers' health information. Through the study of [10], it is highlighted that seafarers who exposed high esteemed work conditions require better follow-up on their health status.

Seafarers are exposed to hard-working conditions when compared to ashore workers [1]. Therefore, it is recommendable to have an immediate follow-up in the provision of health services and continuous health checkups on time. With an understanding of the seafarer's health determinants, it is mandatory to improve prevention methods to treat sailors with accidents and general diseases. However, risk factors and other medical events in seafarers are not well established, and their definition remains hard to handle as well as there are no standardized health monitoring instruments for seafarers [11].

This study proposes a conceptual HO model in the provision of medical assistance to seafarers' health based on the local data repository of the Centro Internazionale Radio Medico (C.I.R.M.). C.I.R.M. is the Italian Telemedical Maritime Assistance Service (TMAS) and represents the organization with the largest number of seafarers assisted at sea worldwide [12].

## 2. Methods

A systematic collection of seafarer's health data from the C.I.R.M. database was done. To have access to the database, C.I.R.M authorities provided consent. Analytical data interpretation was made with text usage, graphs, tables, and other visualization methods to highlight the situation and trends. This will mainly include the collection of relevant datasets from different sources, including TMAS, seafarer's clinics, and shipping companies. The data flow was organized with the support of the World Wide Web Consortium (W3C). Gathering relevant and reliable information for the decision and other data flow can be identified in the W3C.

A preliminary conceptual framework of the seafarer's health observatory (SHO) was developed with the help of an integrative review of the existing seafarer's policy. The review was conducted through libraries of Scopus, PubMed, and Web of Science (WoS). The search string “identifying existing HO systems, and seafarer's health determinants” was identified as 207 studies. English materials with free, full text, which explain the definition and HO functionalities, were included. After the study screening at different phases, ultimately 21 documents were identified that can satisfy the study objective and outcome domains.

In the end, both open and closed questionnaires were distributed to responsible bodies and stakeholders via e-mail to collect their opinions, which can match SHO policies. Based on the elite group recommendations, the model will be revised by having a panel discussion. SHO could be well developed to identify the health gaps in occupational health practice, and the seafarer's health policy extension can help to promote the research evidence of the maritime industry.

A multilayer software framework was developed with individual components that were distributed in different layers to provide individual functionalities. This framework model integrated with the outcomes of the above methods (data collection, study concepts, and responses) was adopted as the base of the conceptual model architecture.

## 3. Results

**Figure 1:**
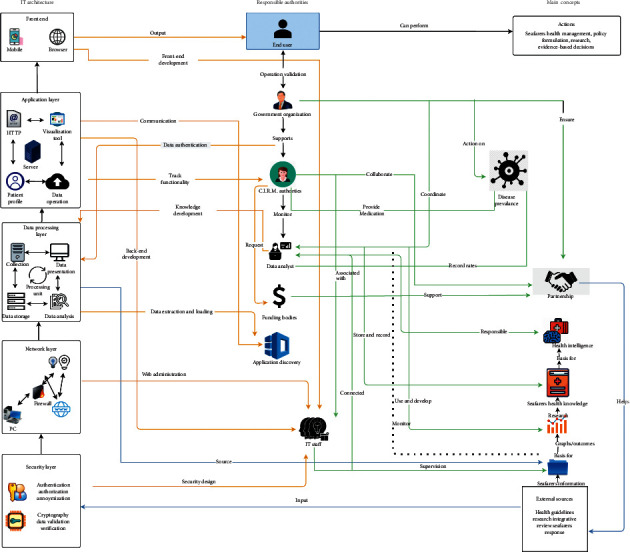
Conceptual framework of seafarers' HO model.

The proposed conceptual HO model is associated with three layers: information technology (IT) layer, stakeholders, or responsible authority layer, and key concept layer is shown in Figure 1.

### 3.1. IT Architecture

IT architecture mainly consists of sublayers such as security [13], networking [14], data processing [15], and application layers [16]. Simple authentication and security layer (SASL) is an authentication framework and provide data security in web protocols. The best practices for efficient data security include a risk-taking approach in data protection with the help of a unique platform. Health data need more security and encryption. Therefore, transport layer security (TLS) was used to facilitate data security and privacy concerns through Internet [13].

The networking layer can provide data routing paths for communication over different computers. The data are transferred to this layer in the form of packets through logical network paths in chronological methods that are controlled by the network layer [14]. The data layer is a description of business needs and goals, which are aligned in a format of immediate transfer to technical specifications. This layer consists of seafarers' health data, which holds total data and can process and transfer through the observatory website to other mobile applications. In general, this layer carries information that can be useful for distinct tools or end users [15].

An application layer can specify the shared information protocols and interfacing of end-user methods in a communication panel. Seafarer's health profiles, risk factors, or pathologies frequency can be visualized from this layer in the form of graphs and statistics when the public audience requests hypertext transfer protocol (HTTP), and this layer can facilitate the user to network services. Therefore, any user can do message transfer, e-mails, or export information from this [16].

Despite this, designing of the web platform could be mandatory to store the health information and policies of seafarers. Health information materials directed to seafarers and shipping companies could be developed. There is significant evidence of nonoptimal health of seafarers, which arises from both the severe work and associated lifestyles. Web platforms can report the latest trends in diseases associated with seafarers and provide brief interventions to assist older seafarers. It also establishes the effect of those interventions on their knowledge, behaviors, health, and wellbeing.

### 3.2. Stakeholders

In this layer, many people from local government, funding bodies, and C.I.R.M. authorities can involve working together. It could make more comfortable the exchange of information to support the decision. An information-sharing section should be established. The analyzed data should provide decision-makers evidence-based information on monitoring, evaluation, and further prioritizing areas. All these stakeholders can work closely together to develop feasible observatory respect to end-user requirements, especially with disease management. IT staff and data analysts are responsible for maximizing the user's experience even on mobile devices. Gathered consent will develop future planning and site maps from the human bodies involved in this layer.

### 3.3. Actions

Research in clinical medicine such as review studies and randomized trials could enhance the health intelligence of seafarers. These medical researchers can explore more opportunities in evidence-based systems. Once the data transferred from the information source, analytical staff conducts data operations to generate knowledge. After that, health intelligence could structure in the evolution of evidence-based statements. Finally, end users can utilize output actions such as policy making, seafarers' health information, and research outcomes.

## 4. Discussion

In this study, we proposed a conceptual framework of the SHO, which is based on IT technologies to help in the design, development, and implementation of the HO procedure. Working in the maritime industry was identified as a high-risk position, and seafarers were always exposed to acute diseases or serious injuries. Especially, CVD is in high number to decide the mortality rates of seafarers [6, 8, 9]. Most of these diseases are probably occupational diseases. Unfortunately, no systematic and detailed data are available on sailors' pathologies. Therefore, insight knowledge of pathologies that affects seafarers' health is still sparse and represents a grey area of maritime medicine.

An HO could provide the model in the careful selection of health quality information on the most critical current and upcoming issues of public health and its determinants. In this study, we proposed SHO that can enhance the health monitor, pathology trends, and other health problems like injuries (i.e., trauma) in seafarers to identify areas for action and possible occupational health information gaps. The importance of establishing an HO in the marine industry can provide evidence-based health information, thereby enabling immediate medical assistance [17].

SHO's can also allow significant health contributions and healthcare policies as well as in delivering services designed explicitly for seafarers. For achieving the goal of the network development with knowledge, information, and surveillance about seafarers with other inter- and intracountries, SHO development is strongly recommended. The SHO can also be benefited from the knowledge gain of the best practice; access statistics on welfare issues, and collaborate with similar motivational institutions.

A collaboration between different organizations and partners should be considered by performing observatories on seafarer's health functions. The group of participants of the observatory on seafarer's health could be the people from maritime medical institutes and TMAS centers [18], national health authorities especially pioneers, managers and supportive analysts, and political bodies. The observatory technical operation will be carried out via a chain of different information and the analytical-based process leading to reporting and dissemination of the health information for decision.

On the other hand, seafarers are the lifeblood of the shipping industry, and every maritime organization can support to ensure seafarers have access to the best facilities and working conditions onboard and inport. By developing HO for seafarers' health, we can improve the health of seafarers.

## 5. Conclusions

The present study was aimed at the development of an observatory on seafarers' health to improve the seafarers' health conditions through monitoring of their health and disease trends, creating a standard instrument (questionnaires), and conducting a risk assessment. The proposed framework was focused on seafarer's health determinants in on-time assessment, analysis, and data interpretation to understand the health pattern and disease trends. Ultimately, the SHO can also propose the strategies contents for noncommunicable disease prevention and control programs in seafarers.

## Data Availability

Data examined for the present study were collected and stored in a closed database by the Centro Internazionale Radio Medico (C.I.R.M.), the Italian Maritime Telemedical Assistance Service (TMAS), in the frame of health surveillance activities performed onboard ships. Data were extracted from the database by C.I.R.M. operators and anonymized before being used for research purposes. C.I.R.M. President is the legal representative of the entity where medical data are kept has authorized access to authors for collecting data of this work.

## References

[1] Jensen O., Charalambous G., Flores A., Baygi F., Canals M., Andrioti D. (2018). Strategies for prevention of non-communicable diseases in seafarers and fishermen: lessons learned. *International Journal of Community & Family Medicine*.

[2] Wilkinson J. (2015). Public health observatories in England: recent transformations and continuing the legacy. *Cadernos de Saúde Pública*.

[3] Pourmalek F. (2012). National health observatories: need for stepped-up action. *The Health*.

[4] Ireland R. (2014). *Liverpool Public Health Observatory Top Tips for healthier providers of health-care in Merseyside and Cheshire Cath Lewis and Alex Scott-Samuel Observatory Report series no 100*.

[5] Observatory A. H. (2016). *Health Observatories*.

[6] Oldenburg M. (2014). Risk of cardiovascular diseases in seafarers. *International Maritime Health*.

[7] Aikaterini D., Vasileios P., Aris C., Kanella Z., Dimitris K., Efthymios K. (2019). Seafarers’ health problems , emergencies , diseases and risk factors . a systematic review of the literature.

[8] Vuksanovic P., Goethe H. (1982). Diseases and accidents among seamen--an international comparison of distribution of diagnoses. *Bulletin of the Institute of Maritime and Tropical Medicine in Gdynia*.

[9] Alves P. M., Leigh R., Bartos G., Mody R., Gholson L., Nerwich N. (2010). Cardiovascular events on board commercial maritime vessels: a two-year review. *International Maritime Health*.

[10] Romero-Paredes M. D. C., Reinoso-Barbero L., González-Gómez M. F., Bandrés-Moya F. (2016). Improving cardiovascular health in Spanish seafarers. *International Maritime Health*.

[11] Lefkowitz R. Y. (2013). *Incidence of Injury and Illness in Merchant Seafarers Incidence of Injury and Illness in Merchant Seafarers Rafael*.

[12] Mahdi S. S., Amenta F. (2016). Eighty years of CIRM. a journey of commitment and dedication in providing maritime medical assistance. *International Maritime Health*.

[13] Turner S. (2014). Transport layer security. *IEEE Internet Computer*.

[14] Pfaff B., Pettit J., Koponen T., Amidon K., Casado M., Shenker S. Extending networking into the virtualization layer.

[15] Hassanalieragh M. Health monitoring and management using internet-of-things (IoT) sensing with cloud-based processing: opportunities and challenges.

[16] Houimli M., Kahloul L., Benaoune S. (2019). Performance analysis of internet of things application layer protocol. *Advances in Intelligent Systems and Computing*.

[17] Ruhl H. A., André M., Beranzoli L. (2011). Societal need for improved understanding of climate change, anthropogenic impacts, and geo-hazard warning drive development of ocean observatories in European Seas. *Progress in Oceanography*.

[18] Westlund K., Attvall S., Nilsson R., Jensen O. C. (2016). Telemedical maritime assistance service (TMAS) to swedish merchant and passengers ships 1997–2012. *International Maritime Health*.

